# Stigmergy co-ordinates multicellular collective behaviours during *Myxococcus xanthus* surface migration

**DOI:** 10.1038/srep26005

**Published:** 2016-05-26

**Authors:** Erin S. Gloag, Lynne Turnbull, Muhammad A. Javed, Huabin Wang, Michelle L. Gee, Scott A. Wade, Cynthia B. Whitchurch

**Affiliations:** 1 The ithree institute, University of Technology Sydney, Ultimo, NSW 2007, Australia; 2 Biotactical Engineering, IRIS, Faculty of Science, Engineering and Technology, Swinburne University of Technology, Hawthorn, VIC 3122, Australia; 3 School of Chemistry, University of Melbourne, Parkville, VIC 3010, Australia

## Abstract

Surface translocation by the soil bacterium *Myxococcus xanthus* is a complex multicellular phenomenon that entails two motility systems. However, the mechanisms by which the activities of individual cells are coordinated to manifest this collective behaviour are currently unclear. Here we have developed a novel assay that enables detailed microscopic examination of *M. xanthus* motility at the interstitial interface between solidified nutrient medium and a glass coverslip. Under these conditions, *M. xanthus* motility is characterised by extensive micro-morphological patterning that is considerably more elaborate than occurs at an air-surface interface. We have found that during motility on solidified nutrient medium, *M. xanthus* forges an interconnected furrow network that is lined with an extracellular matrix comprised of exopolysaccharides, extracellular lipids, membrane vesicles and an unidentified slime. Our observations have revealed that *M. xanthus* motility on solidified nutrient medium is a stigmergic phenomenon in which multi-cellular collective behaviours are co-ordinated through trail-following that is guided by physical furrows and extracellular matrix materials.

The collective behaviour of individuals within self-organised, biological systems often manifests as large-scale pattern networks^1^,^2^. This is true for the active migration of bacterial communities where the collective motion often results in the emergence of dramatic, intricate and often dynamic pattern networks that are particularly striking and beautiful^3^,^4^,^5^. Within higher-order organisms such as social insects and herd migrations, the manifestation of self-organised phenomena is often described by the concept of stigmergy. Stigmergy refers to the self-organisation of multi-agent systems in which an individual leaves persistent cues and signals within the surrounding environment that influence the behaviour of another individual at a later point in time^6^,^7^. In stigmergic self-organisation, these persistent cues and signals can be provided by physical changes to the environment (e.g. trail-following by herd migrations or human traffic) or by chemical signals (e.g pheromone trails by ants). We have recently proposed that stigmergy is an organising principle that is also utilised by bacterial communities to coordinate their multicellular behaviours^8^.

*Myxococcus xanthus* is a model organism for the study of bacterial collective behaviours and self-organisation^9^ whose collective motion has long fascinated scientists. Under favourable conditions, this Gram-negative soil bacterium undergoes a vegetative growth phase characterised by the active migration of hunting packs of cells that prey on neighbouring bacteria^10^. *M. xanthus* has two motility systems, gliding motility and twitching motility, which act independently but can synergistically mediate rapid migration across surfaces. Gliding motility enables the motility of individual cells whereas twitching motility mediates the migration of cell clusters^11^,^12^,^13^. Gliding motility is driven by a complex motility apparatus which is predicted to track along cytoskeletal filaments within the cell^10^,^14^,^15^,^16^,^17^,^18^. The gliding motility apparatus is predicted to interact with a slime that is secreted and deposited on the underlying substratum^19^,^20^,^21^ Cells undergoing gliding motility preferentially travel along this slime which is deposited as trails that appear phase-bright when visualised with phase-contrast microscopy^19^,^21^. For simplicity we will refer to this slime associated with *M. xanthus* gliding motility as “Mx-slime”.

Twitching motility is powered by the extension and retraction of polar type IV pili (T4P)^22^. Twitching motility in *M. xanthus* also requires the production of an exopolysaccharide that we will refer to here as “Mx-EPS”. Mx-EPS has historically been referred to as fibrils, and is thought to provide an anchor for T4P binding to neighbouring Mx-EPS coated cells^23^,^24^. Lipopolysaccharide (LPS), specifically the O-antigen, is also essential for twitching motility in *M. xanthus*, however the function it plays is yet to be determined^25^.

How *M. xanthus* coordinates the actions of the gliding and twitching motility systems and self-organises these collective behaviours is an area of intense interest and is not yet fully understood. One of the limiting factors in studying the motility of *M. xanthus* is that the actively migrating communities tend to be multi-layered and are often grown at an agar-air interface which makes visualisation and tracking of individual cells extremely challenging. The study of individual and collective behaviours during twitching motility by *P. aeruginosa* has been greatly facilitated by the use of an interstitial twitching motility assay in which the cells are inoculated at the interstitial space between a glass coverslip and a thin layer of a nutrient medium solidified with gellan gum^5^,^26^,^27^. Under these conditions, *P. aeruginosa* rapidly migrates using twitching motility and self-organises into an extensive, intricate network of cellular trails. The tendency of cells in the interstitial space to migrate as a monolayer permits the visualisation and tracking of individual cells using phase-contrast microscopy, thereby avoiding potential artefacts introduced with fluorescence microscopy^5^,^26^. As the interstitial twitching motility assay has greatly contributed to our understanding of collective behaviours during twitching motility by *P. aeruginosa*^26^,^28^, we set out to determine if an interstitial assay may aid in the visualisation and analysis of *M. xanthus* motility.

## Results

### Interstitial migration by *M. xanthus* is characterised by extensive pattern formation

**Figure 1:**
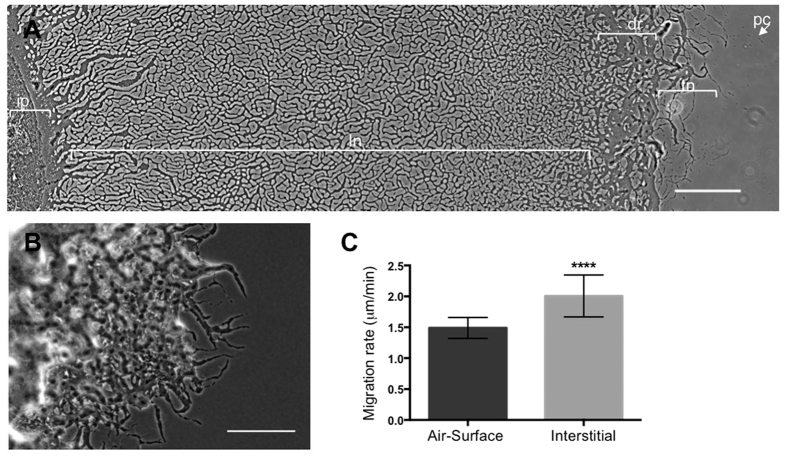
*M. xanthus* interstitial migration is highly organized compared to air-surface migration. Wild-type *M. xanthus* was cultured at interstitial **(A)** and air-surface **(B)** interfaces for 24 h and imaged using phase-contrast microscopy. Scale bar 200 μm. For **(A)** characteristic micro-morphological features are labelled; pc, pioneering cells; tn, tendril network; dr, dense region; ln, lattice network; ip, inoculation point. **(C)** The distances of the leading edges from the inoculation point after 24 h were measured and the migration rate presented as mean ± SD (n = 8). ****P < 0.0001.

**Figure 2:**
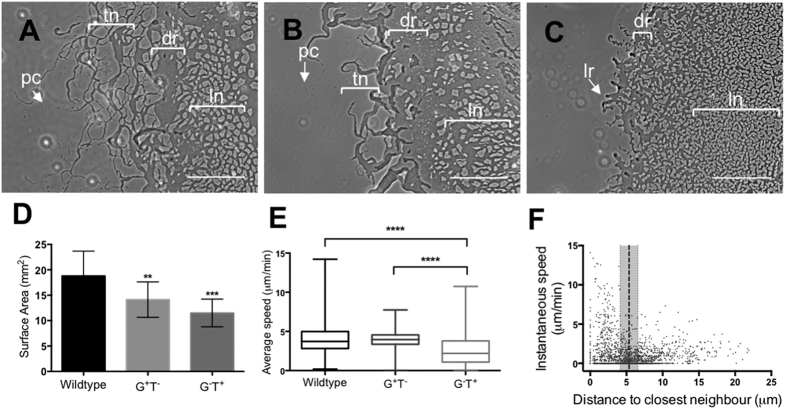
Analyses of *M. xanthus* interstitial migration. Interstitial migration of wild-type **(A)**, G^ + ^T^−^ **(B)** and G^−^T^ + ^**(C**) *M. xanthus* strains was examined using time-lapse phase contrast microscopy (see Supplementary Movie 1). Characteristic micro-morphological features are labelled pc, pioneering cells; tn, tendril network; dr, dense region; ln, lattice network; lr, leading rafts. Scale bar 200 μm. **(D)** Surface areas of *M. xanthus* interstitial colonies after 24 h (mean ± SD for 12 biological replicates for each strain). p-value cf. wild type. **P < 0.01, ***P < 0.001. **(E)** Interstitial migration was examined using time-lapse phase contrast microscopy (see Supplementary Movie 2). Single cells at the leading edge were manually tracked and speeds presented as box and whisker plots of 1074 wild-type cells (n = 3 biological replicates), 324 G^+^ T^−^ cells (n = 3), and 110 G^−^T^+^ cells (n = 9). Box is 25^th^–75^th^ percentiles, line in box is median, whisker limits are minimum and maximum values; ****P < 0.0001. **(F)** Speeds per frame of the time-series (30 s) of G^−^T^+^ cells compared to the distance to the closest neighbour in the direction of motion of the cell at each time point. The dotted line indicates the average length of G^−^T^+^ cells ± SD indicated by the grey bar.

To determine if *M. xanthus* motilities could be examined using an interstitial migration assay, we cultured wild-type *M. xanthus* at the interface between solidified nutrient medium and a glass coverslip. We found that under these conditions wild-type *M. xanthus* actively migrates producing interstitial colonies with characteristic micro-morphological features (Figs 1A and 2A). At the leading edge are single pioneering cells behind which is an interconnected tendril network of cells that precedes a dense, apparently unstructured region. Behind this region is a vast intricate lattice-like network of interconnected cellular trails (Figs 1A and 2A). These micro-morphological features are more intricate and extensive than those typically observed when wild-type *M. xanthus* is cultured at the air-surface interface on solidified nutrient medium (Fig. 1B)^29^. This suggests that *M. xanthus* multicellular communities are capable of more elaborate self-organisation than previously recognised.

We found that the rate of *M. xanthus* migration at the air-surface interface was comparable to that previously reported for *M. xanthus* grown under similar conditions (1.6 μm/min)^29^ whereas migration in the interstitial conditions was significantly faster (Fig. 1C). Interestingly, the micro-morphological patterning manifested more rapidly and more extensively during interstitial migration than at an air-surface interface. Wild-type *M. xanthus* at the air-surface interface displayed very little pattern formation after 24 h with only small tendrils of cells present at the leading edge (Fig. 1B). The characteristic phase-bright trails and associated cellular tendrils were typically only observed at the leading edge of the air-surface colonies after 2–3 days. In comparison, after 24 h interstitial migration manifested as an extensive, intricate micro-morphological patterned structure that included a vast network of interconnected trails of cells (Figs 1A and 2A).

To examine the contribution of gliding and twitching motilities to interstitial migration, *M. xanthus* strains that have been reported to exhibit either only gliding motility (G^+^ T^−^; Δ*aglU*)^30^ or twitching motility (G^−^T^+^; Δ*pilA*)^31^,^32^,^33^, were examined (Fig. 2). Phase-contrast microscopy showed that G^+^ T^−^ interstitial migration was very similar to wild-type but had fewer pioneering cells and the tendrils were thicker and less intertwined (Fig. 2A,B). In contrast, G^−^T^+^ interstitial migration lacked individual pioneering cells and had no tendrils (Fig. 2C). Instead, at the leading edge were rafts of cells behind which was a small, unstructured dense region and an extensive lattice-like network that appeared less organised than wild-type or G^+^ T^−^ (Fig. 2A–C). The micromorphological patterns of cells at the leading edge for all three strains is reminiscent to that observed when these strains are cultured at the air-surface interface^29^. However, these self-organised cellular formations are much more extensive than has been previously reported.

Examination of *M. xanthus* interstitial migration using phase-contrast time-lapse microscopy revealed that the leading edge of wild-type and G^+^ T^−^ consisted of single, well-isolated cells as well as tendrils of cells that appeared to snake out into virgin territory (Fig. 2A,B; Supplementary Movies 1 and 2). The single cells, particularly for G^+^ T^−^, displayed a tendency to loop back upon themselves, forming rings of cells that gradually filled over time with increased cellular traffic (Supplementary Movie 2). For both wild-type and G^+^ T^−^ strains, a network of tendrils formed behind the advancing edge, with this network being considerably more extensive for wild-type, compared to G^+^ T^−^ (Fig. 1A,B; Supplementary Movies 1 and 2). From the higher magnification time-series it was evident that the cells at the leading edge of both wild-type and G^+^ T^−^ were highly confined to trails, leading to the emergence of an interconnected tendril network that appeared stable over time (Supplementary Movie 2). Time-series compressions confirmed that cells tended to remain within trails and traverse identical paths (Supplementary Figs 1A,B and 2A,B). Interestingly, at the leading edge, we often observed the presence of phase-dark trails along which individual cells traversed and appeared to be highly confined (Supplementary Movie 2; Supplementary Fig. 3A,B). Both wild-type and G^+^ T^−^ cells were often observed to turn onto these trails rather than travel through virgin territory (Supplementary Fig. 4; Supplementary Movie 3).

Measurement of the surface areas of the resultant interstitial colonies after culturing for 24 h showed that those of wild-type were significantly larger than either G^+^ T^−^ or G^−^T^+^, which were similar in size to each other (Fig. 2D). Tracking of individual, well-isolated cells (>1 cell-length from closest cell) at the leading edge of wild-type and G^+^ T^−^ revealed that these cells migrated at equivalent average speeds (4.20 ± 1.98 μm/min and 4.00 ± 1.08 μm/min respectively; Fig. 1E), although individual wild-type cells were capable of much higher speeds than G^+^ T^−^ cells as indicated by the range of speeds measured (Fig. 2E). Interestingly, there were about 3-fold fewer single motile cells at the leading edge of G^+^ T^−^ compared to wild-type (Fig. 2E; Supplementary Movie 2). This may account for the more elaborate pattern network that manifests at the leading edge of wild-type compared to G^+^ T^−^ (Fig. 2A,B; Supplementary Fig. 1A,B). Furthermore the rates of the individual wild-type cells observed here (Fig. 1E) is similar to that previously reported for individual wild-type cells migrating at the air-surface interface (4.4 ± 2.2 μm/min)^34^,^35^. Therefore, while individual cells travel at equivalent speeds in both conditions, the greater overall coverage of the community in the interstitial assay (Fig. 1C) suggests that the coordination of the collective behaviours is more efficient at the interstitial interface resulting in greater net outward movement and larger surface area coverage.

In contrast to wild-type and G^+^ T^−^ strains, the leading edge of G^−^T^+^ interstitial migration did not contain any well-isolated pioneering cells, but rather were comprised of large rafts of cells migrating into virgin territory (Fig. 2C; Supplementary Movies 1 and 2). Neighbouring rafts were observed to merge together, leading to the formation of a relatively stable reticulum of cells at the leading edge (Supplementary Figs 1C and 2C; Supplementary Movies 1 and 2). G^−^T^+^ cells have only been reported to display single cell motility during expansion at an air-surface interface when within a maximum separation distance of 0.88 of a cell length (3.5 μm) from its neighbours^29^. It had been hypothesized that this reflects the maximum distance that the T4P can span to bind to the Mx-EPS coating of neighbouring cells for twitching motility to occur^24^,^29^. However, we observed that during interstitial migration, well-isolated G^−^T^+^ cells located in the wake of the leading edge rafts were motile and often appeared to traverse along phase-dark trails (Supplementary Figs 3C and 5; Supplementary Movies 2 and 3). We tracked 110 well-isolated G^−^T^+^ cells and found that these migrated at an average speed of 2.59 ± 1.99 μm/min (Fig. 2E). Surprisingly, 49% of the movements of isolated G^−^T^+^ cells occurred at separation distances greater than the average cell length, with the longest distance being 21.88 μm to the nearest neighbour (Fig. 2F). Interestingly, when these isolated cells came within one cell length from a neighbour, the speed of their movements increased compared to that displayed when at distances greater than one cell length (Fig. 2F). We predict that Mx-EPS may be a component of the phase-dark trails that provides a substrate for T4P binding and subsequent retraction leading to translocation. This would also account for the apparent confinement of migrating cells to these phase-dark trails. The observation that cells increased their speed when within a cell length of a neighbour suggests that direct interaction of cells T4P with neighbouring cells is more effective at facilitating twitching motility (Fig. 2F).

### *M. xanthus* produces a complex extracellular matrix during interstitial migration

During these studies we observed the presence of phase-dark trails at the leading edge of all three strains examined (Supplementary Figs 3–5; Supplementary Movies 2 and 3). These trails appeared to facilitate the trail-following behaviour displayed by the cells in this region. We hypothesised that these trails were comprised of an extracellular matrix (ECM). Therefore in order to gain insight into these trails we visualised potential ECM components by supplementing the nutrient media in our interstitial assay with calcofluor white to stain Mx-EPS^36^,^37^, FM1-43FX or FM4-64FX to stain lipids, or TOTO-1 to stain extracellular DNA (eDNA). *M. xanthus* interstitial migration in the presence of these stains was imaged using wide-field fluorescence microscopy and OMX 3D-structured illumination microscopy (3D-SIM).

**Figure 3:**
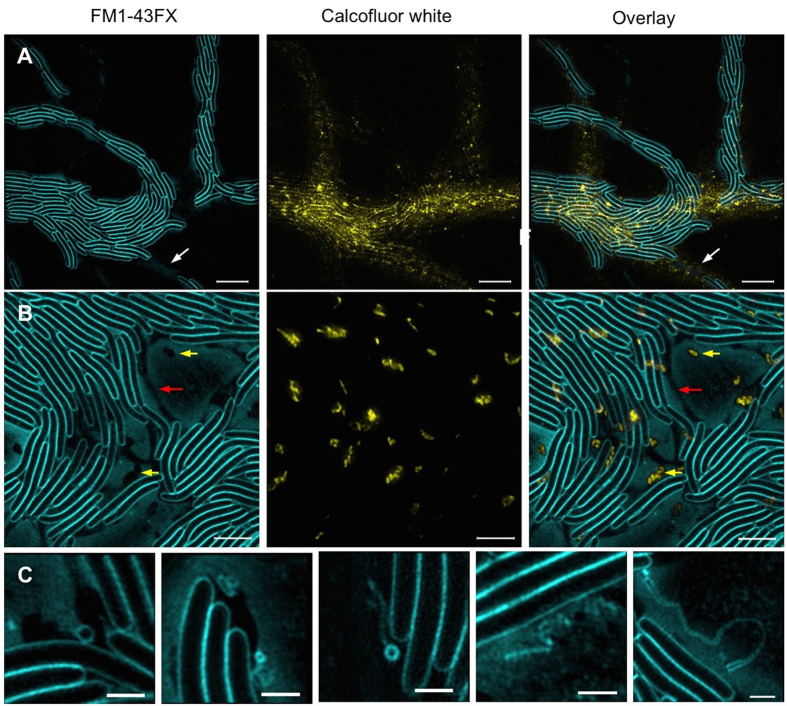
Super-resolution microscopy of the ECM of wild-type *M. xanthus* interstitial migration. Interstitial migration assays of wild-type *M. xanthus* were cultured for 24 h at 30 °C on CYEGG supplemented with FM1-43FX (cyan) to stain lipids and calcofluor white (yellow) to stain Mx-EPS and imaged using 3D-SIM at the leading edge **(A)** and within the lattice network **(B)**. White arrows indicate e-lipid trails at the leading edge. Red arrows indicate regions that occlude the FM1-43FX and calcofluor white. Yellow arrows indicate regions that occlude the FM1-43FX dye but stain with calcofluor white. Scale bar 5 μm in **(A)** and 3 μm in **(B)**. **(C)** Membrane vesicles and structures that may be membrane tubules or membrane fragments were observed within the extracellular milieu. Scale bar 1 μm.

Calcofluor white staining showed that the leading edge of wild-type and G^−^T^+^ contained a fine network of Mx-EPS that coated previously traversed areas (Fig. 3A; Supplementary Figs 6A,B and 7A) and became more globular in nature further back within the lattice network (Fig. 3B; Supplementary Figs 6C and 7B). Calcofluor white staining was not observed in the G^+^ T^−^ *pilA* mutant (Supplementary Fig. 8). As *M. xanthus pilA* mutants do not produce Mx-EPS^38^ this indicates that calcofluor white specifically stains this ECM material, consistent with previous reports^36^,^37^. Furthermore wild-type *M. xanthus* was also found to produce a small amount of eDNA, however it did not appear to play a role during interstitial migration (see Supplementary Results; Supplementary Fig. 9).

Interestingly, extensive quantities of extracellular lipid (e-lipid) material that stained with the FM dyes were also observed (Fig. 3; Supplementary Figs 6–8). At the leading edge, e-lipid staining was present as defined trails, whereas further back within the lattice network it was present as a lawn, filling the open spaces of the network and appearing more concentrated around the periphery of the cellular trails (Fig. 3; Supplementary Figs 6–8). It is possible that this e-lipid material is comprised of small membrane vesicles that could not be resolved using these imaging techniques. Indeed OMX 3D-SIM super-resolution microscopy revealed the presence of larger membrane vesicles (>100 nm) and structures that could be consistent with membrane tubules or fragments within the e-lipid of the trail network (Fig. 3C). The presence of membrane vesicles and outer membrane material deposited on the substratum, have been previously reported in *M. xanthus* biofilms and are predicted to be involved in the exchange of membrane material and signalling molecules between cells^39^,^40^,^41^. However, to our knowledge, this is the first time that such abundant levels of e-lipid have been observed within ECM produced by *M. xanthus*. As we did not see any evidence of cell lysis in our time-series or by eDNA staining, we predict that the e-lipid is produced either through active secretion or is sloughed from the cells. Whether this e-lipid facilitates either gliding motility or twitching motility of *M. xanthus,* is yet to be determined; however, as it is a predominant component of the ECM, we speculate that it may lubricate cellular movement, possibly by promoting a more energetically favourable surface to traverse. It is also possible that at least a component of the e-lipid is the LPS that is required for twitching motility^25^.

**Figure 4:**
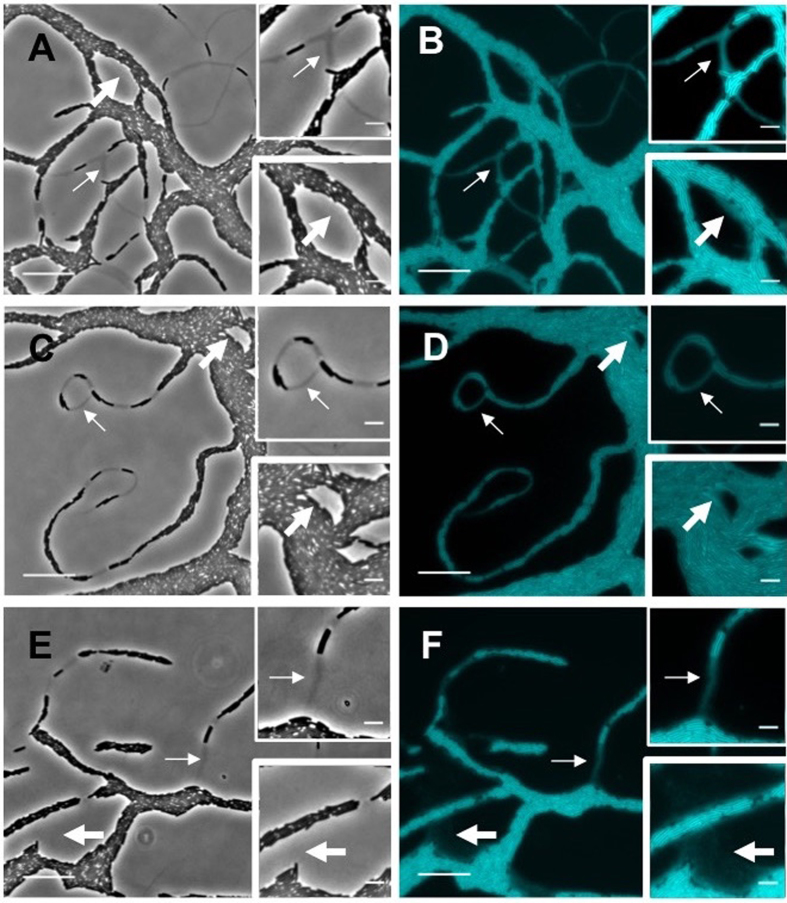
Phase-dark trails are comprised of e-lipid. Interstitial migration assays were cultured for 24 h at 30 °C on CYEGG supplemented with FM1-43FX (cyan) to stain lipids. Time-lapse phase-contrast microscopy was performed at the leading edge of wild-type **(A,B)**, G^+^ T^−^ **(C,D)** and G^−^T^+^ **(E, F)** *M. xanthus* strains (see Supplementary Movie 3), after which the same area was imaged using phase-contrast **(A,C,E)** and fluorescence microscopy **(B,D,F**) showing the presence of phase-dark and e-lipid trails respectively. Thin white arrows indicate phase-dark trails that correlate to e-lipid trails. Thick white arrows indicate areas of diffuse FM1-43FX staining that are not observed as phase-dark trails. Insets show a closer view of the area indicated by the corresponding arrow. Scale bar 20 μm for the main images and 5 μm for the insets.

The e-lipid trails at the leading edge appeared similar to the localisation and morphology of the phase-dark trails that were observed in these regions. To determine if the phase-dark and e-lipid trails were in the same physical location, the medium was supplemented with the lipid dye FM1-43FX and phase-contrast time-lapse microscopy was performed (Supplementary Movie 3), after which the area was imaged using correlative fluorescence microscopy (Fig. 4). The phase-dark trails were found to directly correspond to the e-lipid trails (Fig. 4). Interestingly, e-lipid trails that stained less intensely did not appear phase-dark (Fig. 4), suggesting that a critical concentration of e-lipid is required for these trails to create contrast under phase optics. The ECM produced by wild-type and G^−^T^+^ during interstitial migration contained both e-lipid and Mx-EPS (Fig. 3; Supplementary Figs 6 and 7). However, as G^+^ T^−^ was found to possess little to no Mx-EPS (Supplementary Fig. 8) yet still produced phase-dark trails (Fig. 4C; Supplementary Figs 3 and 4), we conclude that the phase-darkness of these trails was due to the presence of e-lipid.

**Figure 5:**
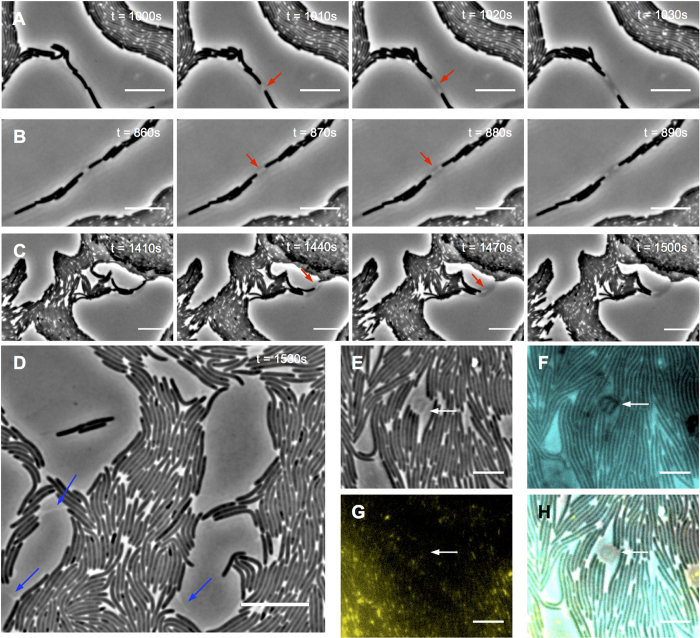
*M. xanthus* cells produce a phase-bright substance that forms clear trails within the e-lipid. Interstitial migration assays were cultured for 24 h at 30 °C and then monitored using phase-contrast time lapse microscopy (see Supplementary Movies 2, 3). Time-series from Supplementary Movie 3 for wild-type (**A**) and G^+^ T^−^ **(B**) *M. xanthus* strains; and from Supplementary Movie 2 for G^−^T^+^ (**C**). Red arrows indicate the presence of a phase-bright substance at the lagging pole of *M. xanthus* cells. (**D**) Interstitial migration of wild-type *M. xanthus* was monitored using high magnification phase-contrast time-lapse microscopy across 30 min (see Supplementary Movie 4). Blue arrows indicate areas where phase-bright regions were observed in the wake of a cell. Time from the start of the time series is indicated in top right hand corner. Scale bar 10 μm. Interstitial migration assays of wild-type *M. xanthus* cultured on nutrient medium supplemented with FM1-43FX (cyan) and calcofluor white (yellow) and imaged using correlative phase-contrast and fluorescent microscopy: phase contrast image (**E**), e-lipid (**F**), Mx-EPS (**G**) and overlay of all 3 channels (**H**). White arrow indicates phase-bright regions that appear to emanate from the lagging poles of a cluster of cells. Scale bar indicates 5 μm.

Both wide-field fluorescence microscopy and OMX 3D-SIM revealed that within the e-lipid filled areas in the lattice network there were many small regions that occluded the lipid stain (Fig. 3B; Supplementary Fig. 6). Co-staining of the ECM with FM1-43FX (lipid) and calcofluor white (Mx-EPS) showed that many of these regions correlated to globular Mx-EPS (Fig. 3B; Supplementary Fig. 6). However, there were also numerous clear regions within the ECM that did not label with either stain (Fig. 3B; Supplementary Fig. 6C). These clear regions also did not stain with the eDNA-specific dye TOTO-1 (Supplementary Fig. 9B). We propose that these clear regions are likely to be composed of another ECM component that is hydrophilic in nature, as it appears to repel the e-lipid. As Mx-slime has been proposed to be a polyelectrolyte gel^21^, it is possible that the clear regions are comprised of Mx-slime that repels e-lipid. Interestingly, we found that when visualised with phase-contrast microscopy, cells travelling along phase-dark trails produced a phase-bright substance that appeared to be associated with the lagging pole of the cells and was often observed when two connecting cells migrated away in opposite directions (Fig. 5A–D; Supplementary Movies 3 and 4). Using correlative phase-contrast and fluorescent microscopy we determined that this phase-bright substance correlated to the clear regions observed in the e-lipid (Fig. 5E–H) and may therefore be comprised of Mx-slime.

### *M. xanthus* forges a furrow network that appear as phase-bright trails

**Figure 6:**
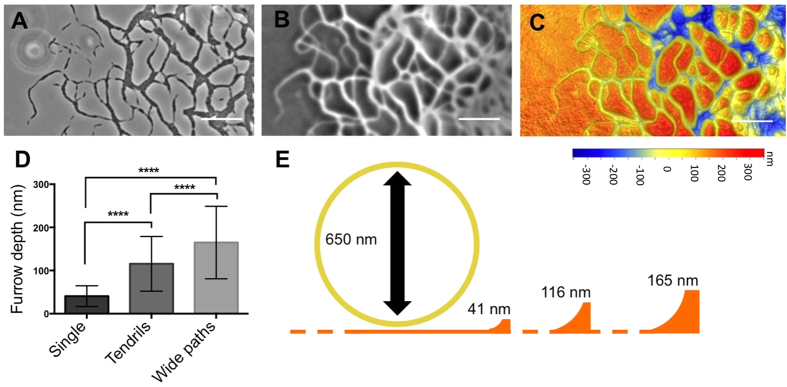
Phase-bright trails are furrows forged during interstitial migration. (**A**) Phase-contrast image of a wild-type *M. xanthus* interstitial migration after 24 h growth at 30 °C and (**B**) the corresponding image of the underlying substratum after the cells had been removed. (**C**) 3D height profile of the underlying substratum depicted in (**B**) imaged using 3D optical profilometry. (**D**) Depths of the furrows forged during interstitial migration in different micro-morphological regions. Only furrows created by a single pioneering cell were measured for the “Single” grouping. Data are presented as mean ± SD. ****P < 0.0001. (**D**) Schematic drawn to scale of the average furrow depths forged during interstitial migration by single cells (41 nm), tendrils of cells (116 nm) and wide paths of cells (165 nm), relative to the average cell height (650 nm).

The emergent pattern formation observed during *M. xanthus* interstitial migration is reminiscent of the behaviours observed during interstitial twitching motility by *P. aeruginosa*^5^,^26^. We have recently determined that *P. aeruginosa* forges an interconnected furrow network in the substratum during interstitial twitching motility, which contributes to the stigmergic self-organisation of the multicellular communities^26^,^42^. To explore if there was a similar furrow network formed during *M. xanthus* interstitial migration, the substratum of interstitial colonies was imaged using phase-contrast microscopy following extensive washing. This revealed the presence of an interconnected network of phase-bright trails that corresponded to the overlying pattern of cells (Fig. 6A,B). These stable phase-bright trails are distinct from the phase-bright substance that we predict to be the Mx-slime which was only visualised transiently (Fig. 5A–D; Supplementary Movies 3 and 4). The topography of the underlying medium was also imaged using 3D optical profilometry. This showed that these phase-bright trails corresponded to an interconnected furrow network (Fig. 6C), which also correlated directly to the micro-morphological pattern of the original cells (Fig. 6A–C).

Correlative live cell time-lapse imaging and optical profilometry revealed that the furrows were deeper in areas with increased cellular traffic (Fig. 6D). We used OMX 3D-SIM to measure the height of live *M. xanthus* cells within the interstitial space. It is evident from these analyses that the furrows forged by the single cells are relatively shallow in comparison to the cell height and would likely offer little guiding capabilities (Fig. 6E). However, with increased cellular traffic the furrows are likely to be of sufficient depth to confine the movements of the cells within the network, thereby guiding their movements (Fig. 6E).

**Figure 7:**
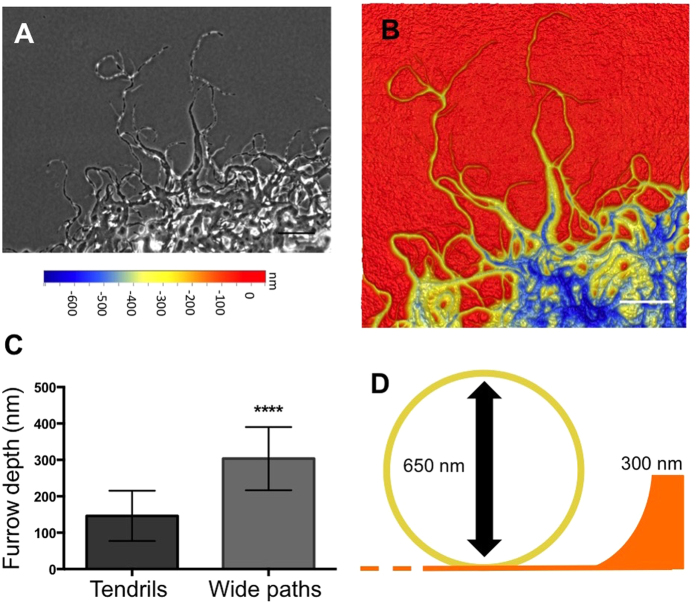
Phase-bright trails that form during *M. xanthus* migration at an air-surface interface are a furrow network. (**A**) Wild-type *M. xanthus* air-surface colonies were cultured for 2 days at 30 °C and imaged using phase-contrast microscopy shows cells in phase-bright trails. (**B**) 3D height profile of the underlying substratum of the colonies depicted in (**A**) imaged using 3D optical profilometry. Scale bars 50 μm. Profilometry depth scales are relative. (**C**) Depths of the furrows forged by air-surface colonies in different micro-morphological regions. Data are presented as mean ± SD. ****P < 0.0001. (**D**) Schematic drawn to scale of the average furrow depths (300 nm) forged by the wide paths of cells during *M. xanthus* air-surface migration, relative to the average cell height (650 nm).

The presence of stable phase-bright trails are also routinely observed at the leading edge during *M. xanthus* air-surface colonies and have been proposed to be composed of Mx-slime^19^,^21^. These trails are thought to aid in the collective behaviour displayed by *M. xanthus*, as cells are observed to preferentially migrate along these phase-bright trails^19^,^43^. In light of our observations that the furrow network formed during interstitial migration appears phase-bright when visualised with phase-contrast microscopy and that the predicted Mx-slime was only transiently observed as a phase-bright substance rather than as stably-deposited trails, we investigated whether the stable phase-bright trails observed at the leading edge of *M. xanthus* air-surface colonies also corresponded to an underlying furrow network. Therefore the topography of the medium underlying wild-type air-surface colonies was imaged using 3D optical profilometry. The phase-bright trails at the leading edge were found to correlate to an interconnected furrow network (Fig. 7A,B). Correlative live cell imaging and optical profilometry revealed that the furrows become deeper in regions with increased bacterial traffic (Fig. 7B,C), similar to what was observed with interstitial migration (Fig. 6B,C). The furrows beneath the air-surface colonies were deeper than those formed during interstitial migration which suggests that the multi-layered nature of the air-surface colony contributes to the creation of deeper furrows. These deep furrows are at least half the height of the cell (Fig. 7D) and so are likely to effectively confine cells to the furrow thereby guiding cell movement. These observations suggest that the phase-bright trails that are routinely observed at the leading edge of *M. xanthus* air-surface colonies likely correlate to an interconnected furrow network that guides bacterial migration.

## Discussion

*M. xanthus* is a soil-dwelling bacterium that assembles into multi-cellular hunting packs that migrate through soil using gliding and twitching motility to prey on other bacterial species. *M. xanthus* has been used for many decades as a model organism to study self-organisation of multicellular bacterial communities. Many of these studies have examined migration at an air-surface interface on solidified nutrient media. However, *M. xanthus* migration in soil is likely to occur in interstitial spaces. In this study we have found that *M. xanthus* displays rapid migration when cultured at the interstitial interface of a coverslip and solidified nutrient medium. Under these conditions *M. xanthus* interstitial migration is characterised by extensive micro-morphological patterning that is considerably more elaborate than that observed at an air-surface interface. The interstitial migration assay described here permits the observation of *M. xanthus* collective behaviours and individual cell movements more readily than at an air-surface interface. Our observations indicate that *M. xanthus* is able to manifest a more highly organised and coordinated form of collective behaviour than previously reported. We utilised this assay to explore *M. xanthus* migration using sophisticated microscopy techniques and have observed several novel phenomena. It is likely that many of these phenomena also occur during migration at an air-surface interface where cells can move in three dimensions but that they are more readily visualised with this interstitial assay in which cells are confined and move as a monolayer in two dimensions and can be visualised at the coverslip with high-resolution microscopy.

In this study we have found that wild-type interstitial colonies were larger than those formed by either G^+^ T^−^ or G^−^T^+^ strains, which were equivalent to one another. Therefore it appears that under the conditions of the interstitial assay, the two motility modes act together to mediate rapid migration, similar to that observed previously at the air-surface interface^29^,^44^.

Interestingly, we have found that during *M. xanthus* wild-type interstitial migration, both gliding and twitching motilities contribute to the migration of well-isolated cells at the leading edge. This contrasts with previous observations of *M. xanthus* air-surface migration where single cells at the leading edge are thought to utilise solely gliding motility and only display twitching motility behind the leading edge where they are in close proximity to neighbouring cells^29^. However, we observed in the interstitial assay that the G^+^ T^−^ strain had fewer single motile cells at the leading edge than wild-type which suggests that twitching motility contributes some single-cell motility by wild-type *M. xanthus* under the conditions of this assay. It is possible that single cells also translocate using twitching motility during migration at an air-surface interface but that this is difficult to observe due to the densely packed and 3-dimensional nature of these communities.

Using the interstitial assay we observed that well-isolated G^−^T^+^ cells are able to migrate along paths comprised of e-lipid, Mx-EPS and possibly Mx-slime. This confirms previous reports where it was observed that single G^−^T^+^ cells were motile when submerged in a high viscosity medium on a polystyrene surface^45^,^46^. Binding of the T4P to the polystyrene was found to initially mediate this single cellular twitching motility, with deposition of Mx-EPS over time further enhancing and coordinating the individual cellular movements^45^. More recently this behaviour has also been observed along carboxymethylcellulose coated microfluidic chambers^47^. However, to our knowledge, our observations are the first report of well-isolated single motile G^−^T^+^ cells migrating under conditions such as solidified nutrient medium that are conducive to complex multicellular collective behaviours by *M. xanthus*.

We hypothesize that the Mx-EPS within the trails facilitates T4P binding and retraction, thereby enabling twitching motility of these cells in the absence of close neighbours. Li *et al*.^24^ have previously hypothesized that the T4P of *M. xanthus* is capable of binding to Mx-EPS deposited within Mx-slime trails and proposed that this would account for the apparent preferential confinement of cells to Mx-slime trails^24^. We propose that during wild-type interstitial biofilm expansion, cells at the leading edge utilise gliding motility during initial exploration into virgin territories and in doing so lay down an ECM trail composed of Mx-slime, e-lipid and Mx-EPS. Continued cellular traffic along these areas results in further deposition of the ECM components, thereby extending and thickening these trails. Once sufficient Mx-EPS has been deposited to enable T4P binding and retraction, single cells are then capable of utilising either gliding motility or twitching motility, or both, with the combined efforts resulting in a more effective exploratory migration of these cells and accounting for the extensive and elaborate pattern network at the leading edge of wild-type cultures undergoing interstitial migration.

In this study we have also shown that during both interstitial and air-surface migration, a network of furrows is forged in the underlying substratum. Our observations suggest that the phase-bright trails associated with *M. xanthus* air-surface colonies that have been previously thought to be Mx-slime trails, may instead be furrows^19^,^21^. We have found that the furrows initially forged by the single pioneering cells are shallow and the furrows become deeper with increased cellular traffic. We predict that the shallow furrows are sufficient to pool the ECM into distinct trails that confine and facilitate the motility of the cells within these areas. This then promotes further cellular traffic along the trails, further reinforcing and deepening the furrows, which also serve to physically confine cells, leading to the continued maintenance of the trail network. We had initially attempted to image the furrow system using atomic force microscopy (AFM), but this proved unsuccessful due to contamination of the AFM tip (Supplementary Fig. 10D) with a sticky substance. This is consistent with the presence of ECM components within the furrows that were resistant to removal by washing with water. Therefore, our observations suggest that during surface migration, *M. xanthus* forges an interconnected furrow network in the substratum that is lined with a bed of ECM. We propose that the furrows and ECM act together to coordinate collective behaviours during *M. xanthus* migration. It is conceivable that as *M. xanthus* hunting packs move through their native soil environment they remodel the soil substratum forming tracks lined with ECM to coordinate their multi-cellular activities.

We have recently proposed that the collective behaviour exhibited during *P. aeruginosa* interstitial twitching motility is coordinated by stigmergic self-organisation^8^,^42^. Interestingly, many of the micro-morphological features of *M. xanthus* interstitial migration described here are reminiscent of the features observed during *P. aeruginosa* interstitial twitching motility including the emergence of large-scale, intricate trail networks^5^,^26^,^42^. This suggests that self-organisation of these actively migrating multicellular communities may occur through similar mechanisms. Indeed, our observations suggest that ECM components and furrows produced by vanguard cells facilitate and guide subsequent cellular movements during *M. xanthus* migration on solidified nutrient media. We propose therefore that the self-organised multicellular behaviours that occur during *M. xanthus* surface migration are coordinated, at least in part, through stigmergy.

## Materials and Methods

### Bacterial strains and media

Bacterial strains used in this study include the *M. xanthus* wild-type DK1622 strain^11^, the G^+^ T^−^ strain DK10410 (Δ*pilA*)^31^,^32^,^33^, and the G^−^T^+^ strain MxH1777 (Δ*aglU*)^30^. Both DK10410 (Δ*pilA*) and MxH1777 (Δ*aglU*) were verified by complementation during their initial characterisations^30^,^31^,^32^,^33^. Strains were maintained on CYE medium (1% casitone, 0.5% yeast extract, 4 mM MgSO_4_.7 H_2_O) with 10 mM MOPs buffer (pH 7), solidified with 1.5% agar at 30 °C.

### Migration assays

Air-surface migration assays were performed on CYE medium solidified with 0.8% gellan gum (MP Biomedicals) (CYEGG) supplemented with 10 mM MOPs in 35 mm petri dishes. The surface was inoculated in the centre of the plate with the strain of interest, placed in a humidified chamber and incubated at 30 °C for 48 h. Interstitial migration assays were performed on glass microscope slides coated with CYEGG. The centre of the slide was inoculated with the strain of interest, gently covered with a glass coverslip so that the cells were sandwiched between the CYEGG and the coverslip and incubated at 30 °C for 24 h. Air-surface and interstitial assays were inoculated using a sterile inoculation loop containing a match-head sized scraping of cells from the agar plate on which the *M. xanthus* strains were maintained and gently touching this to the surface of the solidified CYEGG. CYEGG was supplemented as indicated with 5 μg/mL FM1-43FX or FM4-64FX; 5 μg/mL calcofluor white; 1 μM TOTO-1 (Life Technologies) or 100 KU/mL DNaseI (D5025, Sigma Aldrich). For 3D optical profilometry, cells were removed prior to imaging by placing the dish on an orbital platform with 1 mL water at 100 rpm overnight. Excess liquid and loose cells were then removed by washing and samples were fixed with 1 mL of 4% paraformaldehyde to kill any residual cells.

### Microscopy

Phase-contrast and time-lapse microscopy of interstitial migration were performed as previously described^26^, with the environmental control chamber maintained at 30 °C. Wide-field fluorescence microscopy was performed on a DeltaVision personal DV inverted microscope (Applied Precision Inc., GE Healthcare) fitted with a xenon light source and 512 × 512 Cascade EMCCD camera (Photometrics) or a Nikon TI inverted research microscope fitted with an Lumencor LED light source and a 1 K × 1 K Cascade EMCCD camera (Photometrics). 3D-SIM was performed on a DeltaVision OMX-Blaze^TM^ imaging system (Applied Precision Inc., GE Healthcare) as previously described^26^,^48^. AFM and 3D optical profilometry imaging and analysis were performed as previously described^26^,^42^.

### Image Processing

Images were processed in FIJI v.1.0^49^ or IMARIS v.7 (Bitplane AG, Zurich). Movements of single cells from Supplementary Movies 2 and 4 were tracked manually with the TrackMate plugin in FIJI. Migration rates of air-surface and interstitial migration were calculated from the distance traversed from the inoculation point after 24 h.

### Statistics

Data are presented as mean ± S.D. One-way ANOVA with a Tukey’s post-hoc test and Student’s t-test was used to compare data sets. Analyses were performed using GraphPad Prism v.6 (Graphpad Software). Statistical significance was determined using a p-value < 0.05.

## Additional Information

**How to cite this article**: Gloag, E. S. *et al*. Stigmergy co-ordinates multicellular collective behaviours during *Myxococcus xanthus* surface migration. *Sci. Rep.* **6**, 26005; doi: 10.1038/srep26005 (2016).

## Supplementary Material

Supplementary Information

Supplementary Movie 1

Supplementary Movie 2

Supplementary Movie 3
