# Supplementary material for: Stigmergy co-ordinates multicellular collective behaviours during Myxococcus xanthus surface migration
Source: Sci Rep. 2016 May 26;6:26005. doi: 10.1038/srep26005 (PMC4881031; doi:10.1038/srep26005)
Supplement: Supplementary Information [file srep26005-s1.pdf]

Stigmergy co-ordinates multicellular collective behaviours during *Myxococcus xanthus* surface migration

Erin S. Gloag[a], Lynne Turnbull[a], Muhammad A. Javed[b], Huabin Wang[c], Michelle L. Gee[c], Scott A. Wade[b] and Cynthia B. Whitchurch[a,]*

Supplementary Results

Extracellular DNA is not required for interstitial gliding motility

We have recently shown that extracellular DNA (eDNA) is required for biofilm development by *P. aeruginosa* [1] and facilitates the coordinated migration of *P. aeruginosa* cells during interstitial twitching motility [2]. It has been shown that eDNA is required for *M. xanthus* fruiting body formation, where it helps maintain the architecture for the 3D structure [3]. To determine if eDNA was also present during wild-type *M. xanthus* interstitial migration, the medium was supplemented with TOTO-1, a cell impermeable nucleic acid dye. This revealed that there was little to no eDNA at the leading edge (Supplementary Fig. 9A). Within the lattice network, the eDNA was observed as concentrated foci of eDNA (Supplementary Fig. 9B).

To determine if eDNA is required during *M. xanthus* interstitial migration, the medium was supplemented with DNaseI. These assays showed that there was no difference in the overall micro-morphology of interstitial migration in the presence or absence of the enzyme (Supplementary Fig. 9C, D), nor was there a difference in the surface area coverage (Supplementary Fig. 9E). Therefore, eDNA is not required for *M. xanthus* interstitial migration.

Supplementary References

1 Whitchurch, C. B., Tolker-Nielsen, T., Ragas, P. C. & Mattick, J. S. Extracellular DNA required for bacterial biofilm formation. *Science* **295**, 1487 (2002).

2 Gloag, E. S. *et al.* Self-organization of bacterial biofilms is facilitated by extracellular DNA. *Proc. Natl. Acad. Sci. U.S.A.* **110**, 11541-11546 (2013).

3 Hu, W. *et al.* DNA Builds and Strengthens the Extracellular Matrix in *Myxococcus xanthus* Biofilms by Interacting with Exopolysaccharides. *PLoS One* **7**, e51905 (2012).

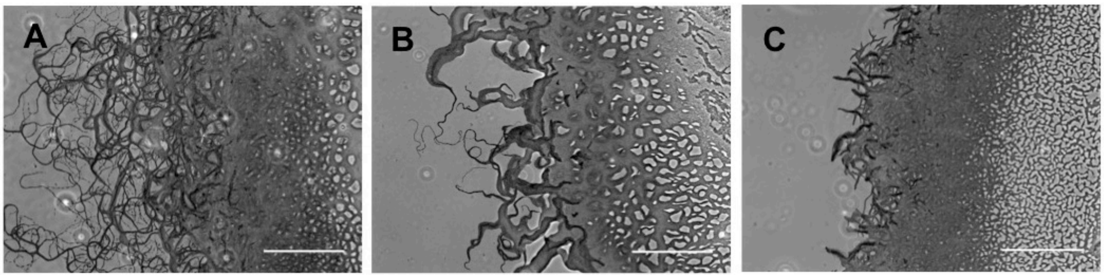

Supplementary Figure 1. Maintenance of the micro-morphological patterns during *M. xanthus* interstitial migration. *M. xanthus* interstitial migration was captured using low magnification phase-contrast time-lapse microscopy (see Supplementary Movie 1). The time-series were compressed to visualise the cellular traffic during interstitial migration of wild-type **(A)**, $G^{+}T^{-}$ **(B)**, and $G^{-}T^{+}$ **(C)** *M. xanthus* strains. Darker regions indicate areas of increased cellular traffic. Scale bar 200µm

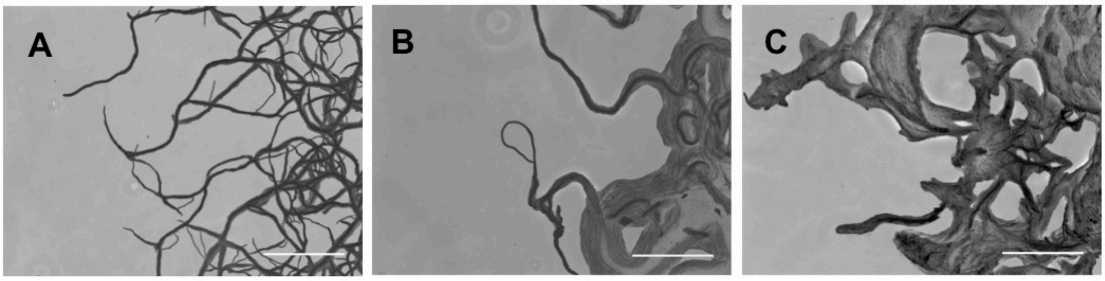

Supplementary Figure 2. Maintenance of the micro-morphological patterns at the leading edge during *M. xanthus* interstitial migration. *M. xanthus* interstitial migration was captured using high magnification phase-contrast time-lapse microscopy (see Supplementary Movie 2). The time-series were compressed to visualise the cellular traffic during interstitial migration of wild-type **(A)**, $G^{+}T^{-}$ **(B)**, and $G^{-}T^{+}$ **(C)** *M. xanthus* strains. Darker regions indicate areas of increased cellular traffic. Scale bar 50μm.

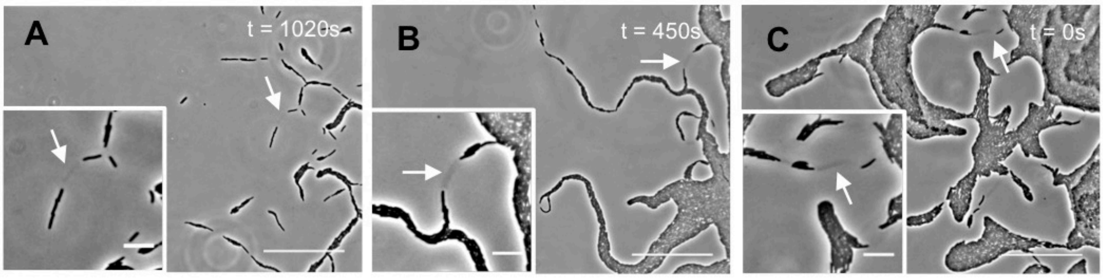

Supplementary Figure 3. Phase-dark trails are observed at the leading edge during *M. xanthus* interstitial migration. From high magnification time-series (see Supplementary Movie 2) phase-dark trails were observed that the leading edge of wild-type **(A)**, $G^{+}T^{-}$ **(B)** and $G^{-}T^{+}$ **(C)** interstitial migration, indicated by the white arrows. Inset is a magnified view of the area indicated by the white arrow. Scale bar 50µm for the main images and 10µm for the insets. The time from the start of the time series (see Supplementary Movie 2) is indicated in the top right hand corner.

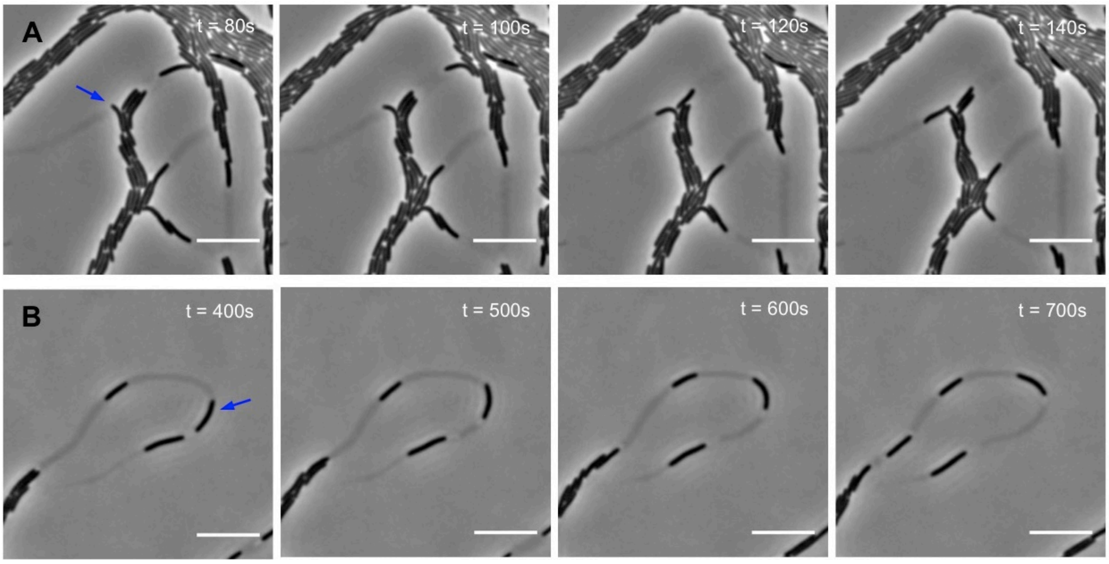

Supplementary Figure 4. *M. xanthus* cells turn onto phase-dark trails. Interstitial migration assays were cultured for 24 h at 30°C and the leading edge was monitored across 30 min using high magnification time-lapse phase-contrast microscopy (see Supplementary Movie 3). Both *M. xanthus* wild-type **(A)** and $G^{+}T^{-}$ **(B)** cells were frequently observed to turn onto the phase-dark trails when encountered during motility (blue arrow). The time from the start of the time series (see Supplementary Movie 3) is indicated in the top right hand corner. Scale bar 10μm.

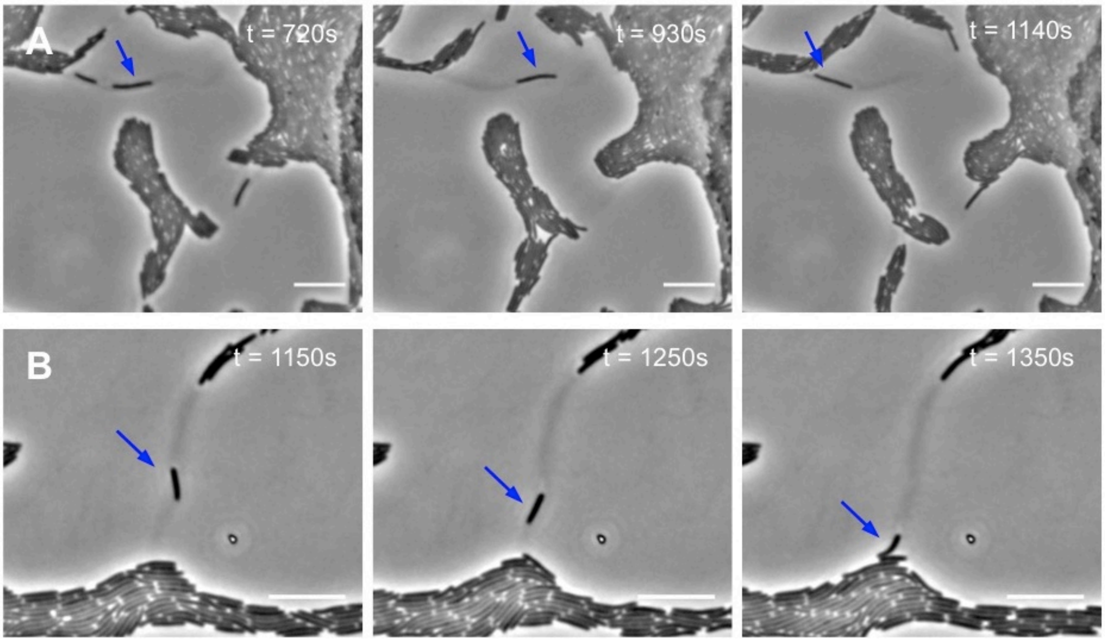

Supplementary Figure 5. Movement of single isolated $G^{-}T^{+}$ cells. Interstitial migration assays of *M. xanthus* $A^{-}S^{+}$ were cultured for 24 h at 30°C after which the leading edge was monitored for 2 h (see Supplementary Movie 2) and 30 min (see Supplementary Movie 3) using high magnification time-lapse phase-contrast microscopy. Motility of isolated single cells was observed within the wake of the advancing rafts; the blue arrow indicates one such cell observed in Supplementary Movie 2 **(A)** and Supplementary Movie 3 **(B)**. The time from the start of the time series is indicated in the top right hand corner. Scale bar 10μm.

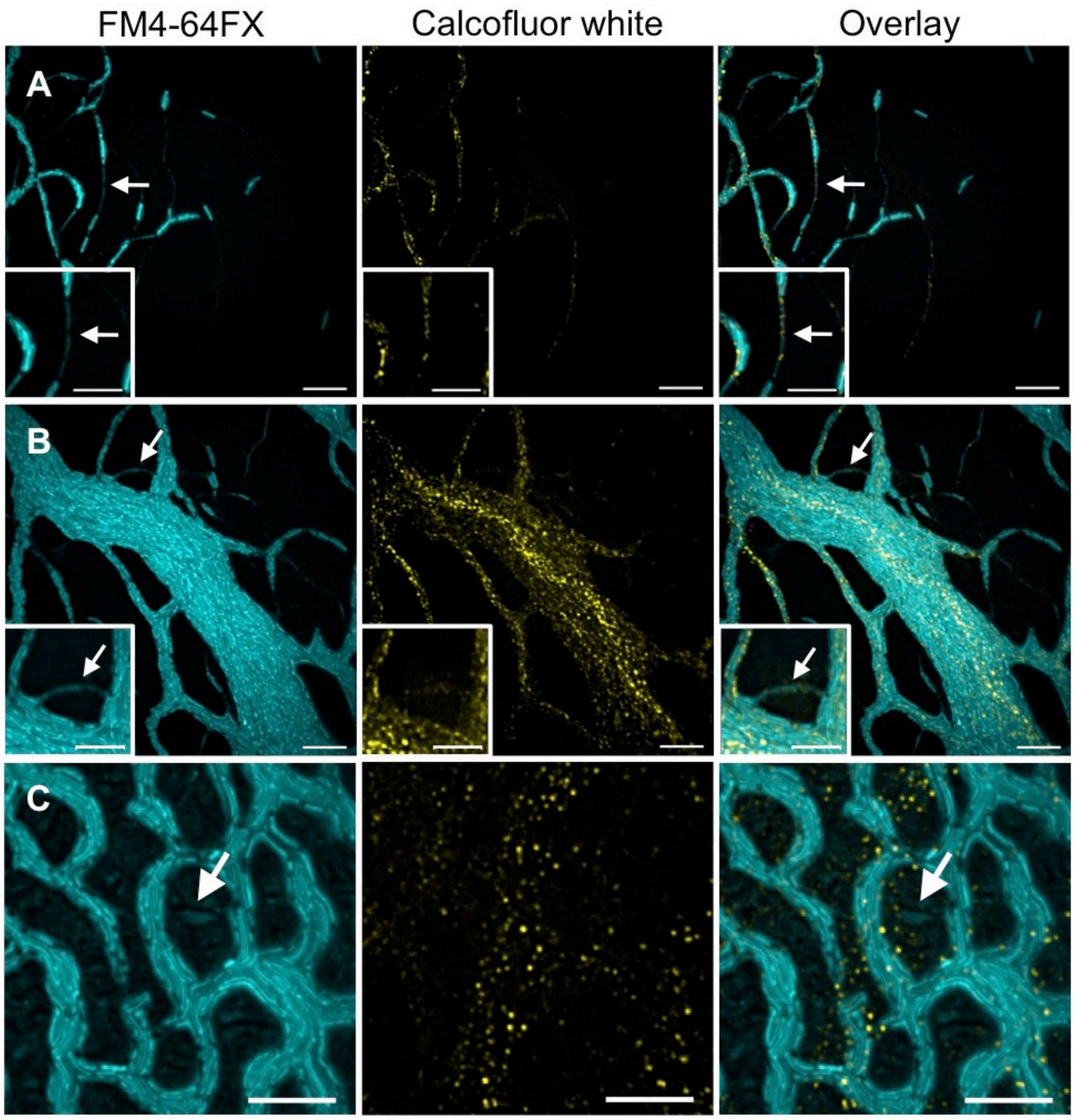

Supplementary Figure 6. The ECM of wild-type *M. xanthus* contains S-EPS and e-lipid. Interstitial migration assays of wild-type *M. xanthus* were cultured for 24 h on CYEGG supplemented with FM4-64FX (cyan) to label lipids and calcofluor white (yellow) to label the S-EPS and imaged using wide-field fluorescent microscopy and imaged at the leading edge **(A)**, the tendril network **(B)** and the lattice network **(C)**. Labels indicate the channels used; left panel: FM4-64FX, middle panel: calcofluor white and right panel: overlay of FM4-64FX and calcofluor white channels. Small white arrows indicate e-lipid trails. Large white arrows indicate trails within areas of e-lipid that have occluded the FM4-64FX dye. Insets are

a magnified view of the regions indicated by the arrow. Scale bar 15μm for the main images and 10μm for the insets.

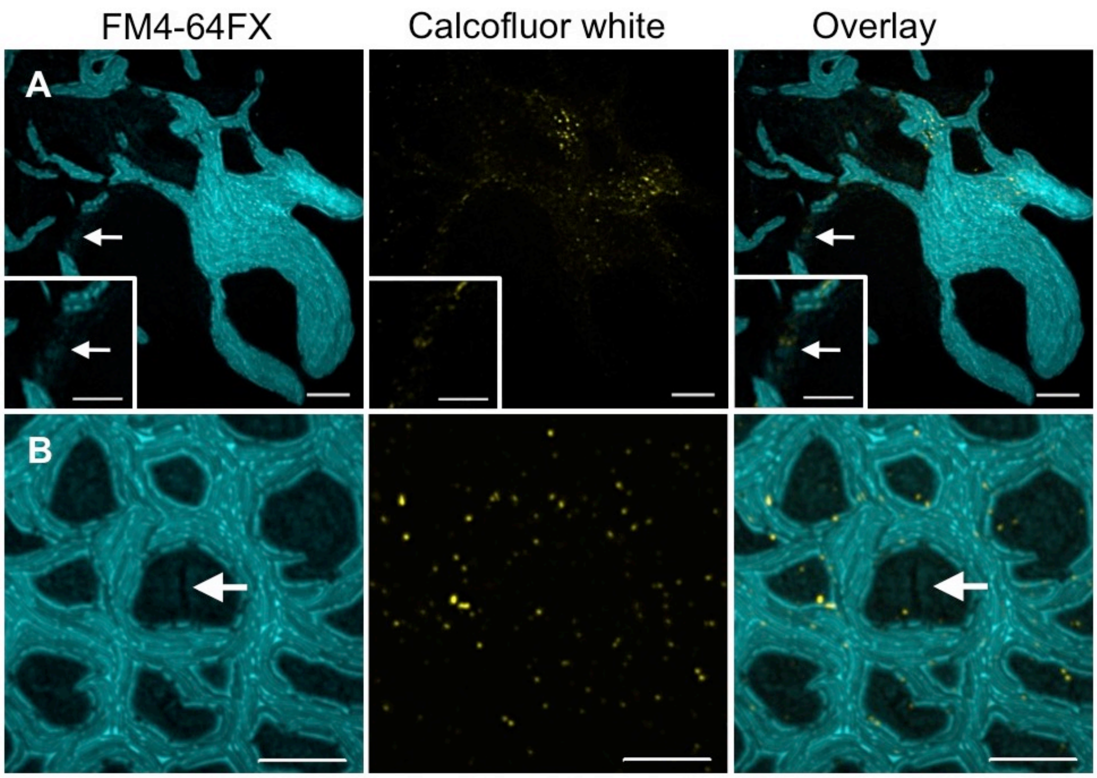

Supplementary Figure 7. The ECM of *M. xanthus* $G^{-}T^{+}$ contains S-EPS and e-lipid. Interstitial migration assays of *M. xanthus* $G^{-}T^{+}$ were cultured for 24 h on CYEGG supplemented with FM4-64FX (cyan) to label lipids and calcofluor white (yellow) to label the S-EPS and imaged using wide-field fluorescent microscopy and imaged at the leading edge **(A)** and the lattice network **(B)**. Labels indicate the channels used; left panel: FM4-64FX, middle panel: calcofluor white and right panel: overlay of FM4-64FX and calcofluor white channels. Small white arrows indicate e-lipid trails. Large white arrows indicate trails within areas of e-lipid that have occluded the FM4-64FX dye. Insets are a magnified view of the regions indicated by the arrows. Scale bar 15µm for the main images and 10µm for the insets.

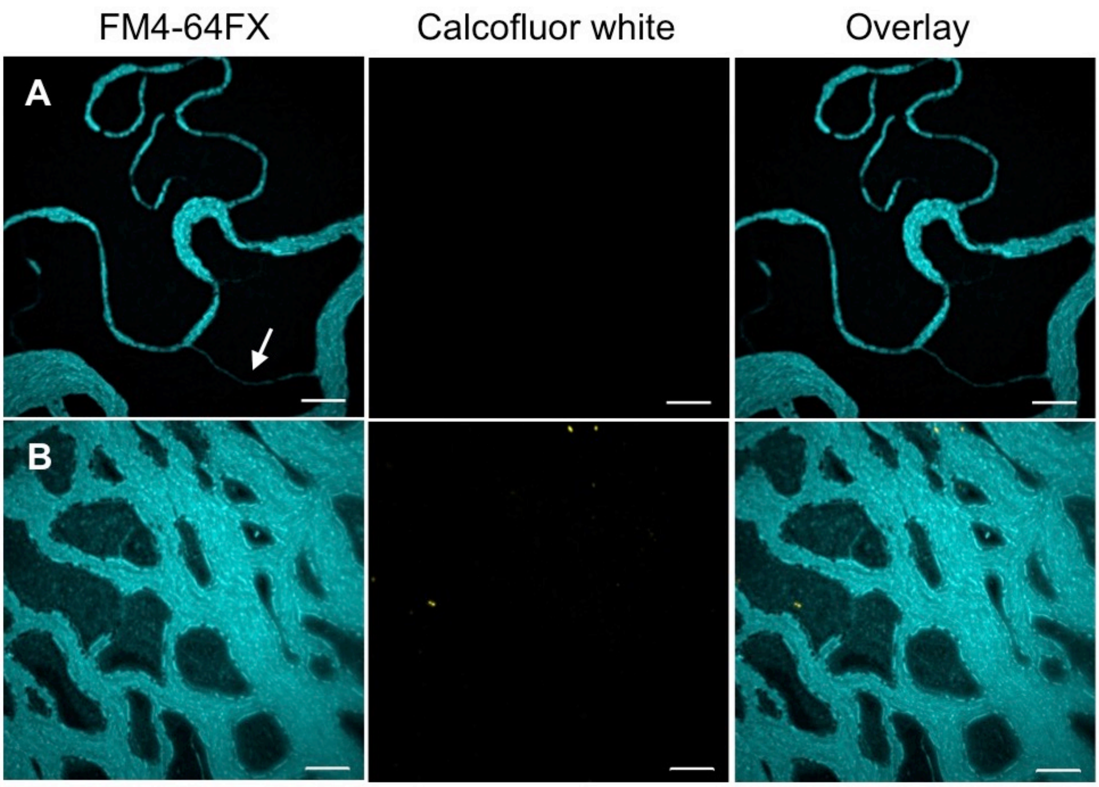

Supplementary Figure 8. The ECM of *M. xanthus* G^+T^- contains e-lipid but no S-EPS. Interstitial migration assays of *M. xanthus* G^+T^- (Δ*pilA*) were cultured for 24 h on CYEGG supplemented with FM4-64FX (cyan) to label lipids and calcofluor white (yellow) to label the S-EPS and imaged using wide-field microscopy at the leading edge **(A)** and the lattice network **(B)**. Labels indicate the channels used; left panel: FM4-64FX, middle panel: calcofluor white and right panel: overlay of FM4-64FX and calcofluor white channels. White arrow indicates e-lipid trails. Scale bar 15μm.

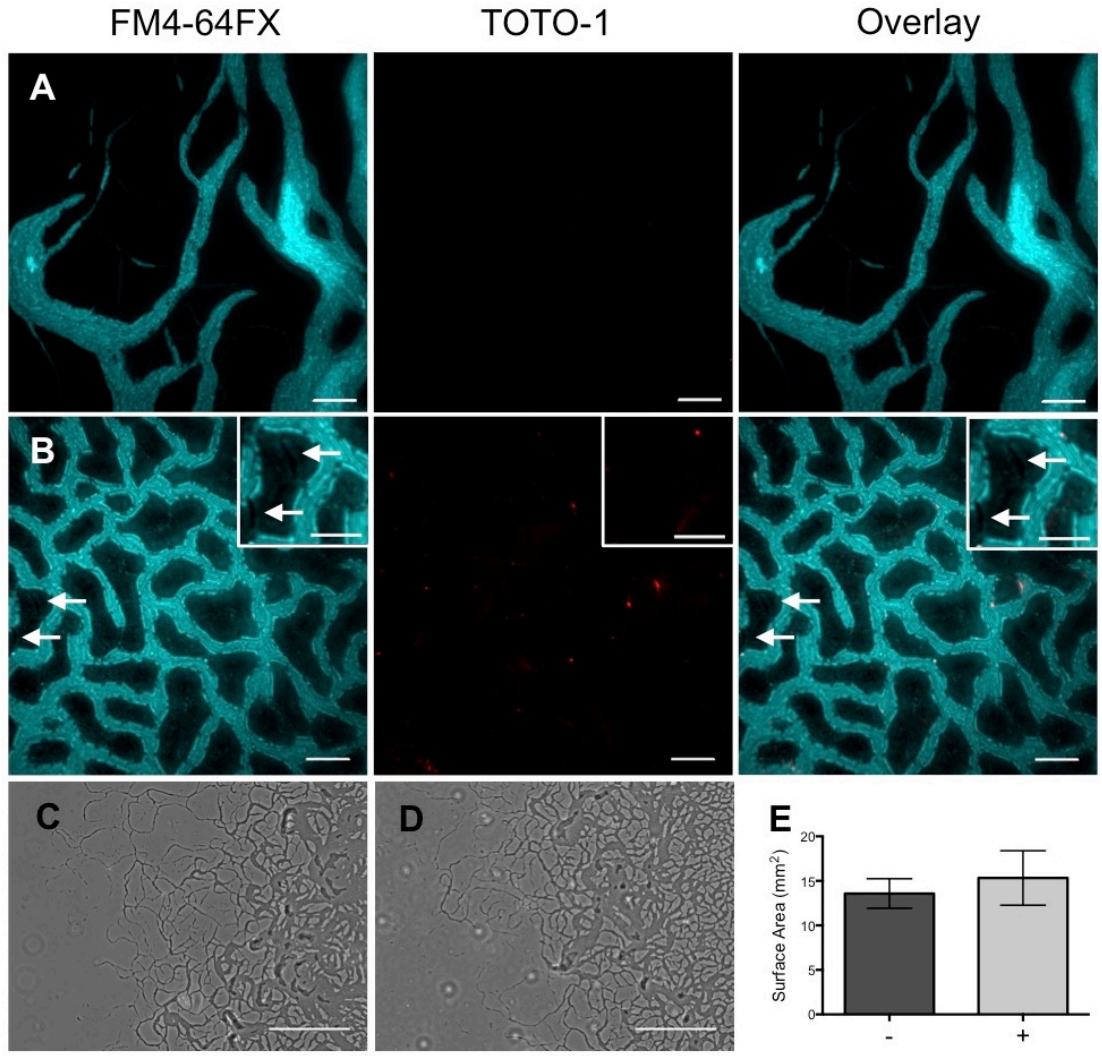

Supplementary Figure 9. A small amount of eDNA is present in the ECM of *M. xanthus* but is not required for interstitial migration. Interstitial migration assays of wild-type *M. xanthus* were cultured on CYEGG supplemented with FM4-64FX (cyan) to label lipids and TOTO-1 (red) to label eDNA and imaged using wide-field fluorescent microscopy and imaged at the leading edge **(A)**, and the lattice network **(B)**. Labels indicate the channels used; left panel: FM4-64FX, middle panel: TOTO-1 and right panel: overlay of FM4-64FX and TOTO-1 channels. White arrows indicate trails within areas of e-lipid that have occluded the FM4-64FX dye indicating that these regions do not correlate to eDNA. Insets are a magnified view of the regions indicated by the arrows. Scale bar 15μm for the main images and 10μm for the insets. Interstitial gliding motility assays in **(C)** the absence (-) and **(D)**

presence (+) of DNaseI. Scale bar 200µm. **(E)** Surface area of the resulting *M. xanthus* interstitial colony after 24 h presented as mean ± SD (n = 12).

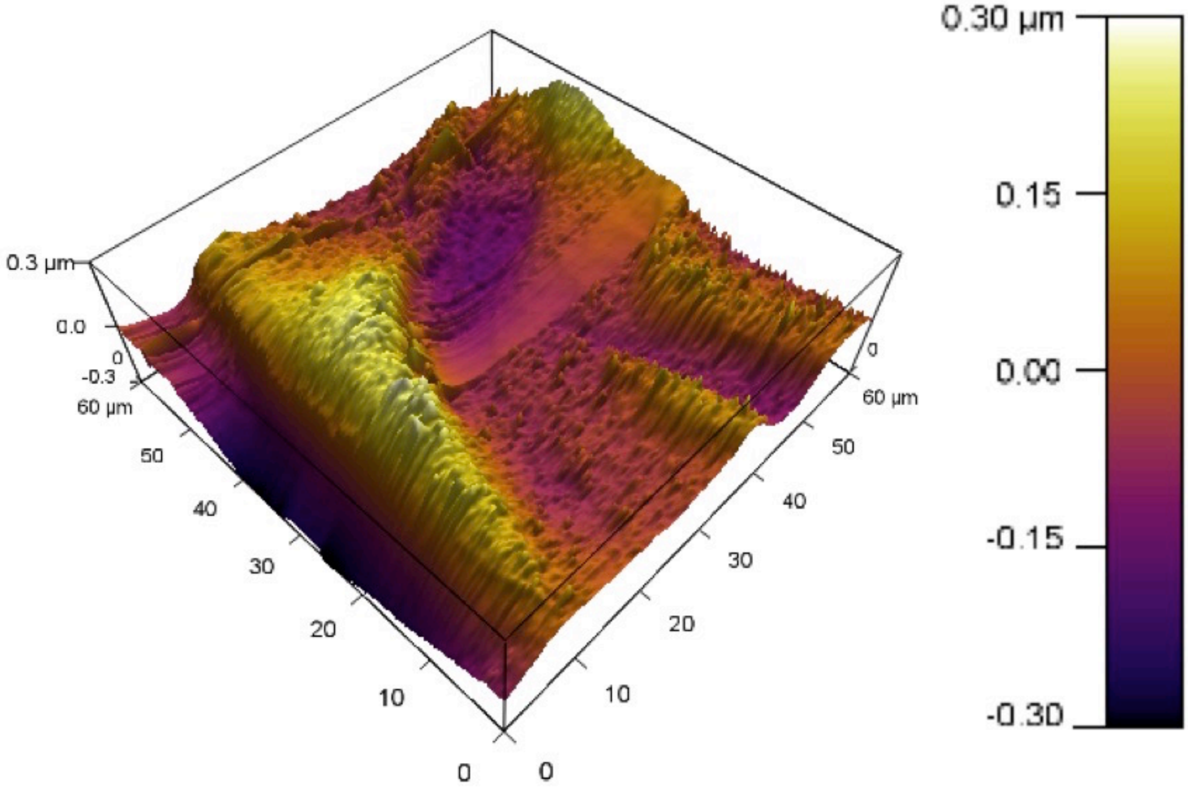

Supplementary Figure 10. Furrow network underlying the tendril network formed during wild-type interstitial migration. Samples were prepared as described for optical profilometry and imaged using atomic force microscopy (AFM). Height scale is relative. The parallel ridges spanning the image is a consequence of contamination of the tip due to ECM remaining on the surface following washing.

Supplementary Movie Legends

Supplementary Movie 1. Overview of *M. xanthus* interstitial migration. *M. xanthus* interstitial migration was monitored using phase contrast time-lapse microscopy for 4 h with a capture rate of one frame every 60 s. Label in the top left hand corner for each panel indicates the *M. xanthus* strain depicted; left panel: wild-type, middle panel: $G^{+}T^{-}$, right panel: $G^{-}T^{+}$. Time is indicated in the lower left hand corner. Scale bar 200 μm.

Supplementary Movie 2. Advancement of the leading edge during interstitial migration. The leading edge of wild-type *M. xanthus* during interstitial migration was monitored using phase contrast time-lapse microscopy for 2 h with a capture rate of one frame every 30 s. Label in the top left hand corner for each panel indicates the *M. xanthus* strain depicted; left panel: wild-type, middle panel: $G^{+}T^{-}$, right panel: $G^{-}T^{+}$. Time is indicated in the lower left hand corner. Scale bar 50 μm.

Supplementary Movie 3. *M. xanthus* cells preferentially follow phase-dark trails during interstitial migration. Phase contrast time-lapse microscopy of the leading edge of *M. xanthus* interstitial migration where phase-dark trails were observed. Capture rate was one frame every 10 s for 30 min. Label in the top left hand corner for each panel indicates the *M. xanthus* strain depicted; left panel: wild-type, middle panel: $G^{+}T^{-}$, right panel: $G^{-}T^{+}$. Time is indicated in the lower left hand corner. Scale bar 20 μm.

Supplementary Movie 4. Appearance of phase-bright regions during *M. xanthus* interstitial migration. Phase contrast time-lapse microscopy was performed within the lattice network of wild-type interstitial migration for 30 min with a capture rate of one frame every 10 s. Time is indicated in the lower left hand corner. Scale bar 10 μm.
